# Behavioral Differences Across Theta Burst Stimulation Protocols. A Study on the Sense of Agency in Healthy Humans

**DOI:** 10.3389/fnins.2021.658688

**Published:** 2021-07-09

**Authors:** Giuseppe A. Zito, Yulia Worbe, Jean-Charles Lamy, Joel Kälin, Janine Bühler, Samantha Weber, René M. Müri, Selma Aybek

**Affiliations:** ^1^Movement Investigation and Therapeutics Team, ICM, Inserm U1127, CNRS UMR 7225, UM75, Sorbonne University, Paris, France; ^2^Department of Neurophysiology, Saint-Antoine Hospital, APHP.6 – Sorbonne University, Paris, France; ^3^Department of Neurology, Inselspital, Bern University Hospital, University of Bern, Bern, Switzerland; ^4^Perception and Eye Movement Laboratory, Department of Neurology and Biomedical Research, Inselspital, Bern University Hospital, University of Bern, Bern, Switzerland

**Keywords:** theta burst stimulation, 30 Hz, 50 Hz, sense of agency, behavioral differences

## Abstract

**Background:**

Theta burst stimulation (TBS) is a non-invasive brain stimulation method. Various stimulation protocols have been proposed, for instance, stimulation at 50 Hz with pattern at 5 Hz, or at 30 Hz with pattern at 6 Hz. To identify better stimulation parameters for behavioral applications, we investigated the effects of 50-Hz continuous TBS (cTBS) on the sense of agency (SoA), and compared them with a previously published study with 30-Hz cTBS.

**Methods:**

Based on power analysis from a previous sample using two applications of 30-Hz cTBS, we recruited 20 healthy subjects in a single-blind, Vertex-controlled, randomized, crossover trial. Participants were stimulated with one application of 50-Hz cTBS over the right posterior parietal cortex (rPPC), a key area for agency processing, and the vertex, in a random order. A behavioral task targeting the SoA was done before and after stimulation. After controlling for baseline differences across samples, we studied the effect of stimulation in the two protocols separately.

**Results:**

Compared to the previously published 30-Hz protocol, 50-Hz cTBS over the rPPC did not reveal significant changes in the SoA, similar to sham Vertex stimulation.

**Conclusion:**

One application of 50-Hz cTBS was not sufficient to elicit behavioral effects, compared to two applications of 30-Hz cTBS, as previously described. This may be due to a mechanism of synaptic plasticity, consolidated through consecutive stimulation cycles. Our results are relevant for future studies aiming at modulating activity of the rPPC in cognitive domains other than agency, and in patients affected by abnormal agency, who could benefit from treatment options based on TBS.

## Introduction

Theta burst stimulation (TBS) is a non-invasive brain stimulation (NIBS) method, based on repetitive transcranial magnetic stimulation (rTMS), consisting of triplets of electromagnetic pulses delivered to the cortex at a specific pattern within the theta range, i.e., 4–7 Hz (Suppa et al., 2016; Jannati et al., 2019). Even though the effects of TBS have been well documented, the optimal parameters have been object of debate (Gamboa et al., 2011). The original protocol applies 50 Hz triplets at 5 Hz, and is known to have inhibitory effects when 600 pulses are delivered continuously over 40 s [continuous TBS (cTBS)], or excitatory ones when 8-s breaks are introduced every 2 s [intermittent TBS (iTBS)] (Huang et al., 2005). It has also been proposed that repeated applications of TBS may induce opposite effects, depending on the duration of the intervals between them. One study, for instance, reported a prolongation of inhibitory effects after two applications of cTBS at 15-min interval from each other (Nyffeler et al., 2006b), whereas others found that two cTBS sessions spaced at 2–5 min suppress the effects of a single session (Gamboa et al., 2011). Studies in patients have also shown positive outcomes of TBS repeated several times a day in hemispatial neglect (Koch et al., 2012; Cazzoli et al., 2015), tinnitus (Plewnia et al., 2012), or addiction (Zhao et al., 2020) (for a review, see Lefaucheur et al., 2014). In parallel to the widely-used 50-Hz protocol, a modified version of TBS has been developed, where 30-Hz triplets are sent at 6 Hz (Nyffeler et al., 2006b). Recent research has suggested that repeated applications of 30-Hz cTBS induce strong behavioral changes when applied over the posterior parietal cortex (PPC) (Fu et al., 2015; Nyffeler et al., 2019; Zito et al., 2020a). Only one study compared these protocols, but focused on motor evoked potentials (MEP), showing a preference for the latter (Goldsworthy et al., 2012a). No study has examined whether the original 50-Hz protocol induces similar behavioral effects to the modified 30-Hz one, leaving the optimization of the stimulation parameters an open question.

In a previous study, we demonstrated, in healthy subjects, the effectiveness of two applications of 30-Hz cTBS over the right PPC (rPPC) on the sense of agency (SoA) (Zito et al., 2020a), the sense of being in control of our actions (Haggard, 2017). In this study, we sought to investigate whether one application of the original 50-Hz cTBS induces similar behavioral changes with the same behavioral paradigm. The ultimate goal of our study is to provide a recommendation on which of these two protocols induces better behavioral changes targeting the SoA in the PPC.

## Materials and Methods

### Ethical Approval, Power Analysis, and Sample Description

Ethical approval was obtained by the Ethics Committee of Canton Bern, Switzerland, and the study aligned with the Declaration of Helsinki. All participants gave written informed consent prior to the study.

Based on the previous sample (Zito et al., 2020a), we used power analysis to calculate the minimal sample size for a two-way repeated-measure analysis of variance (rmANOVA), assuming Cohen’s *d* = 0.86 and a correlation among repeated measures as the minimal Pearson’s correlation coefficient between each pair of repeated measures, i.e., 0.39. This analysis was done with G^∗^Power 3.1.9.7 (University of Düsseldorf, DE) (Faul et al., 2007). For α = 0.05 and a desired power of 0.8 (Cohen, 1988a), the minimal required sample size was 15. We then included 20 healthy volunteers to align with the previous sample.

The 20 subjects (10 males, 10 females, mean age: 27.2 ± 3.3 years) received 50-Hz cTBS over the rPPC or Vertex stimulation. The previous sample (Zito et al., 2020a) consisted of 20 healthy volunteers (10 males, 10 females, mean age: 26.3 ± 3.2 years) who received two consecutive applications of 30-Hz cTBS over the rPPC or Vertex stimulation. Inclusion criteria were: age over 16 years and normal or corrected-to-normal visual acuity. Exclusion criteria were: presence of metal clips in the body, implanted medical devices, past neurological surgery, history of epilepsy, alcohol or drug abuse, pregnancy, or breastfeeding.

### Study Design and Task Description

**FIGURE 1:**
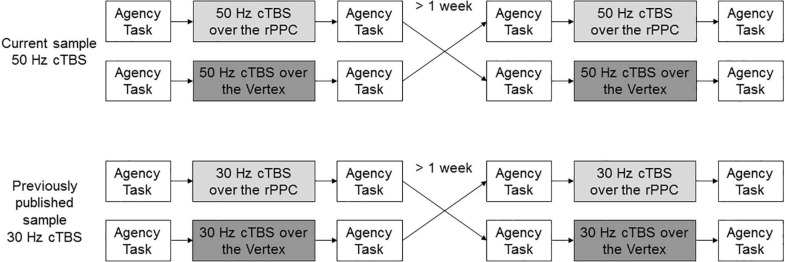
Study design. Participants underwent 50 Hz over the rPPC and Vertex, randomly assigned, in a cross-over fashion. The results were compared with a previously published sample undergoing 30-Hz cTBS in a similar manner.

The study design was a single-blind (participants only), Vertex-controlled, randomized, cross-over trial, whose results were *a posteriori* compared with the previously published sample (Figure 1).

The experiment always took place in the early afternoon. Each group underwent a cTBS session over the rPPC and a Vertex session, crossed in random order with a wash-out period of at least 1 week to eliminate potential residual effects, as suggested in Nyffeler et al. (2006b), Goldsworthy et al. (2012a), and Zito et al. (2020a). Due to the parallel arms, the order of the stimulation was generated with two independent random seeds electronically implemented, for the 50-Hz and the 30-Hz protocol separately, and assigned at inclusion in the study. The previously acquired sample also underwent a session of iTBS, randomly assigned, which is not considered for the purpose of this study. In each session, the participants first performed a behavioral task, then received either cTBS over the rPPC or Vertex stimulation, and immediately after were asked to perform the task again. At the end, they were screened for potential side effects of rTMS, including nausea, pain, or trouble concentrating (Rossi et al., 2009).

**FIGURE 2:**
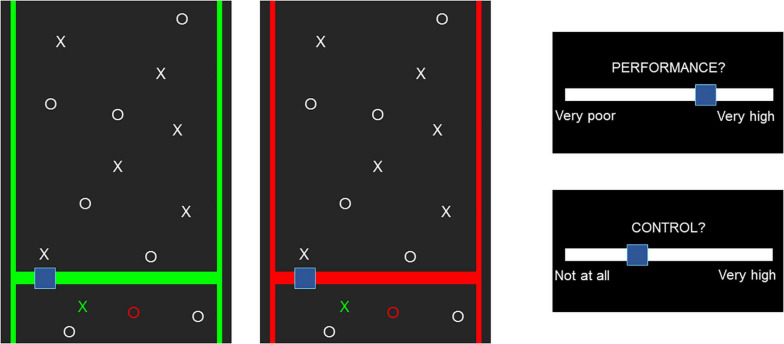
The behavioral task. If the blue box hit the targets, they turned green, and if distractors were hit, they turned red. If not touched, targets and distractors remained white. After each round, participants were asked to rate their performance and sense of control.

The effects of cTBS were quantified with a behavioral task targeting the SoA (Figure 2) (Metcalfe and Greene, 2007), which was already shown to be sensitive to modulation by means of 30-Hz cTBS (Zito et al., 2020a). Participants were placed in front of a computer screen, adjusted in height to align with the level of their eyes, and placed their dominant hand on a response pad. They were shown a pattern of 13 targets and 13 distractors moving down at constant speed and were instructed to catch the targets while avoiding the distractors. They moved a box right or left by clicking on the buttons of the pad. When hit, targets turned green and distractors turned red. Each repetition lasted for 15 s, after which participants judged their performance (JoP) and their sense of control over the game [judgment of agency (JoA)] on a Likert scale from −5 (low JoP/JoA) to +5 (high JoP/JoA). Three game modes were present: Baseline, Magic, and Turbulence. In Baseline, the box moved exactly as instructed by the participant. In Magic, the hitbox increased for targets, but not for distractors, resulting in enhanced performance by hitting the targets more efficiently. In Turbulence, the sense of control and the actual performance were decreased by random movements of the box in 25% of the clicks. Moreover, the frame of the game was green or red. Participants were told that a green color indicated an easy game, and a red color a hard game. However, the difficulty did not change across colors; 54 rounds were played in random order, 22 in Baseline, 16 in Turbulence, and 16 in Magic. Half of them were played with a red frame and the other half with a green frame. Before each round, a fixation cross appeared for 2 s. A two-trial practice session of the task was always performed prior to the experiment. The task was programmed in Matlab R2017b (MathWork Inc., United States) using the Psychtoolbox (Brainard, 1997).

### TMS Protocols

To deliver TMS, a MagPro x100 Device connected to a 70-mm figure-of-eight coil (MC-B70 Butterfly) was used (MagVenture Inc., United States).

For every participant, the resting motor threshold (rMT) was determined with the relative frequency method (Groppa et al., 2012). In this method, single pulses at different intensities are sent to the motor cortex of the participant, while the contralateral hand rests on a pillow. The rMT was defined as the lowest intensity at which five out of 10 consecutive pulses elicited visible twitches in the abductor pollicis brevis (APB) muscle (McConnell et al., 2001; Stokes et al., 2005). To achieve this, the coil was first positioned approximately over M1, visually identified by the examiner, with an orientation of 45° to the mid-sagittal plane (Brasil-Neto et al., 1992; Janssen et al., 2015). The coil position and the stimulation intensity were then varied until reliable twitches were seen. The exact location of M1 was determined by varying the position of the coil until the twitches’ amplitude was maximal. Once the location of M1 was fixed, the intensity was adjusted according to an adaptive staircase fashion, i.e., the stimulator intensity was decreased after trials in which an APB response was present on more than 5/10 pulses, and increased when it was less than 5/10 (Stokes et al., 2005), with a step size of 1% of the maximum stimulator output. The rMT was always measured by the same examiner.

**FIGURE 3:**
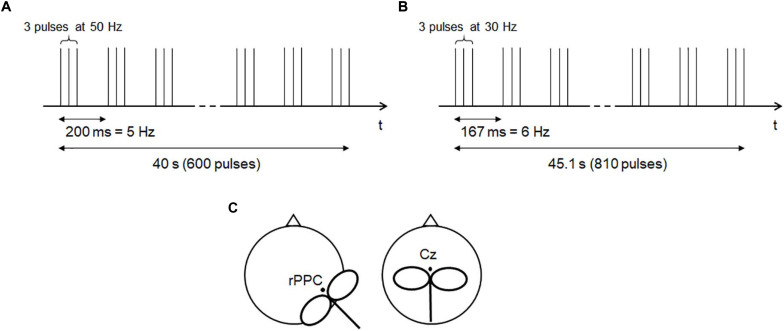
Theta burst stimulation protocols. **(A)** The 50-Hz protocol (Huang et al., 2005). **(B)** The 30-Hz protocol, adapted from Nyffeler et al. (2006b). **(C)** Schematic of coil positioning.

For cTBS stimulation, an intensity of 80% of the rMT was selected (Nyffeler et al., 2006b; Zito et al., 2020a). This choice was made to align the stimulation intensity to the previously published sample. In order to modulate the SoA, the MNI coordinates 62 −34 30 were used as target area, according to Zito et al. (2020a). These coordinates were transformed into EEG 10-20 coordinates by the Münster T2T-Converter (Steinsträter et al., 2002), a method that projects MNI coordinates on the scalp, expresses them in relation to the position of the standard EEG electrodes, and matches them with the actual head of the participant. This minimized potential bias due to head size differences across subjects. Considering C4 the closest reference electrode, and the distance between C4 and C6 as the unit, the rPPC calculated with this method was located at 0.4 units inferior to C4, and 0.1 units posterior to it. The coil was held at 45° toward the contralateral forehead, with the handle pointing posteriorly (Figure 3C). As control region, Vertex stimulation was applied over the interhemispheric fissure above Cz, with the coil’s handle orthogonal to the forehead (Figure 3C). This region was chosen because it is not known to be associated with agency processing. Participants were not aware of the differences in the expected effects from the stimulated regions, and this ensured proper blinding.

The 50-Hz cTBS protocol (Huang et al., 2005) (Figure 3A) was applied in 200 triplets at 50 Hz, with stimulation pattern at 5 Hz, for 40 s (600 pulses). The previously published 30-Hz protocol (Nyffeler et al., 2006a) (Figure 3B) was applied in 270 triplets at 30 Hz, with stimulation pattern at 6 Hz, for 45.1 s (810 pulses). Triplets consisted of biphasic pulses with an anterior–posterior/posterior–anterior (AP-PA) induced current direction (Davila-Pérez et al., 2018). The 50-Hz cTBS protocol was applied once, whereas the 30-Hz one was applied twice with a 15-min interval, according to Nyffeler et al. (2006b), Cazzoli et al. (2012), and Koch et al. (2012). Both protocols were administered by the same examiner.

### Data Analysis

Data analysis was performed with STATISTICA 8.0 (StatSoft Inc., United States).

As we were not interested in the effect of difficulty, the green and red games were pulled together. The SoA was extracted from the median JoP and JoA in each repetition, and expressed as summary agency score (SAS) (Metcalfe et al., 2010):

$$S\,A\,S_{\%}=\left(J\,o\,P_{B\,a\,s\,e\,l\,i\,n\,e}-J\,o\,A_{B\,a\,s\,e\,l\,i\,n\,e}\right)-\left(J\,o\,P_{M\,o\,d\,e}-J\,o\,A_{M\,o\,d\,e}\right)$$

where Mode refers either to Magic or Turbulence. The SAS is designed as a double comparison, where JoA is compared to JoP, and Mode is compared to Baseline. As such, the SAS captures the accuracy of people’s JoA over their perceived performance, in the conditions in which they are not fully in control compared to Baseline, and has shown to be a valid measure of the SoA (Metcalfe et al., 2010; Miele et al., 2011; Zito et al., 2020a). In general, the more negative the SAS is, the lesser control is perceived. A SAS close to zero indicates that the participant feels in control.

Group homogeneity in age, rMT, and time-of-day when the measurement took place was studied with independent sample *t*-tests. Group homogeneity in the SAS prior to stimulation was studied with rmANOVA with within-subject factor STIMULATION (rPPC pre, Vertex pre) and between-subject factor GROUP (50, 30 Hz), for Turbulence and Magic separately.

The specific effects of the two protocols were studied with rmANOVA with within-subject factors STIMULATION (rPPC, Vertex) and TIME (pre, post), for 50 and 30 Hz separately. The effect size of the interaction STIMULATION × TIME was calculated as Cohen’s *d*, for the two protocols separately (Cohen, 1988b).

Correlations between the differences post–pre cTBS and age, rMT, and time-of-day were computed with Pearson’s correlation coefficients. For all statistics, a two-sided significant threshold of *p* < 0.05 was applied. *Post hoc* comparisons were performed with Tukey HSD-corrected *t*-tests (Tukey, 1953).

## Results

No adverse effects were reported after the 50-Hz protocol. After the 30-Hz protocol, five subjects reported mild headache, and five reported mild trouble concentrating, for a few minutes after the stimulation.

When studying the group homogeneity, no differences were found in age [*t*(38) = 0.86, *p* = 0.393], rMT [*t*(38) = 0.26, *p* = 0.799], and time-of-day in rPPC [*t*(38) = 1.04, *p* = 0.305], and Vertex [*t*(38) = 0.34, *p* = 0.733]. RmANOVA on the SAS prior to TBS revealed no main effect of GROUP in Turbulence [*F*(1,38) = 0.10, *p* = 0.757] and Magic [*F*(1,38) = 0.01, *p* = 0.921], no main effect of STIMULATION in Turbulence [*F*(1,38) = 0.13, *p* = 0.722] and Magic [*F*(1,38) = 0.54, *p* = 0.466], and no effect of interaction in Turbulence [*F*(1,38) = 0.02, *p* = 0.887] and Magic [*F*(1,38) = 0.26, *p* = 0.614], indicating no statistical differences between groups or stimulation types before TBS.

**FIGURE 4:**
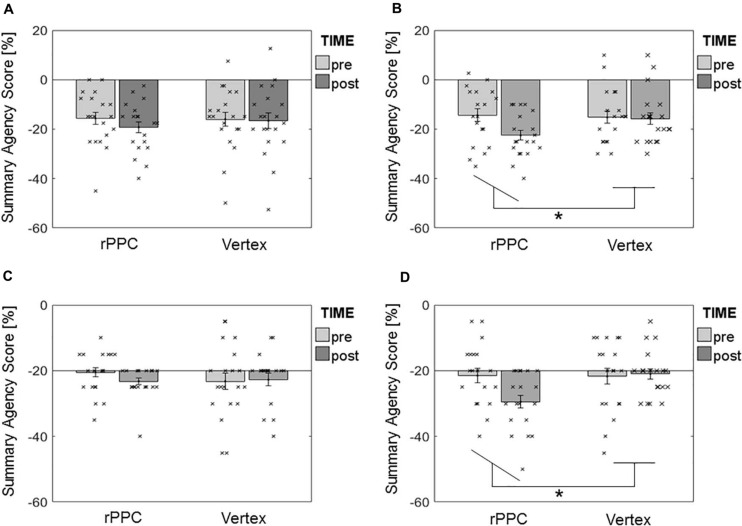
Results of rmANOVA on the summary agency score during Turbulence. **(A)** 50-Hz protocol, Turbulence. **(B)** 30-Hz protocol, Turbulence. **(C)** 50-Hz protocol, Magic. **(D)** 30-Hz protocol, Magic. Bars represent mean values ± SE of the mean. Dots represent the single participants’ values. * Depicts significant STIMULATION × TIME interaction.

The results of rmANOVA on the specific effects of cTBS during Turbulence showed no effect of interaction STIMULATION × TIME in the 50-Hz protocol [*F*(1,19) = 0.80, *p* = 0.381, *d* = 0.41] (Figure 4A), in contrast with a significant interaction in the previously published 30-Hz one [*F*(1,19) = 4.55, *p* = 0.046, *d* = 0.98] (Figure 4B). For the latter, Tukey HSD-corrected *t*-tests showed that the SAS_Turbulence_ significantly decreased from (m = −14.4 ± sd = 11.4)% to (−22.5 ± 8.9)% after the application of 30-Hz cTBS over the rPPC (*p* < 0.05). The 95% confidence intervals (CIs) of the post–pre differences in the SAS_Turbulence_ after cTBS over the rPPC were CI_50Hz_ = (−6.86 −0.39)% and CI_30Hz_ = (−13.48 −2.77)%. No main effect of STIMULATION was found in any of the protocols ([*F*(1,19) = 0.38, *p* = 0.545] for 50 Hz and [*F*(1,19) = 3.54, *p* = 0.075] for 30 Hz). No main effect of TIME was found in the 50-Hz protocol [*F*(1,19) = 2.08, *p* = 0.166], in contrast to a significant effect in the 30-Hz one [*F*(1,19) = 10.35, *p* = 0.005]. The latter was most likely driven by the effect of TIME in the rPPC stimulation (Figure 4B).

Similarly, cTBS during Magic showed no effect of interaction STIMULATION × TIME in the 50-Hz protocol [*F*(1,19) = 0.60, *p* = 0.449, *d* = 0.36] (Figure 4C), in contrast with a significant interaction in the previously published 30-Hz one [*F*(1,19) = 4.59, *p* = 0.045, *d* = 0.98] (Figure 4D). For the latter, Tukey HSD-corrected *t*-tests showed that the SAS_Magic_ significantly decreased from (−0.7 ± 5.8)% to (−4.4 ± 4.4)% after the application of 30-Hz cTBS over the rPPC (*p* < 0.05). The 95% CIs of the post–pre differences in the SAS_Magic_ after cTBS over the rPPC were CI_50Hz_ = (−3.33 0.58)% and CI_30Hz_ = (−7.15 −0.09)%. Neither a main effect of STIMULATION ([*F*(1,19) = 0.37, *p* = 0.551] for 50 Hz and [*F*(1,19) = 3.44, *p* = 0.079] for 30 Hz), nor of TIME ([*F*(1,19) = 0.37, *p* = 0.551] for 50 Hz and [*F*(1,19) = 4.14, *p* = 0.056] for 30 Hz) was found in any of the protocols.

No correlation between the difference post–pre cTBS in the SAS and age, rMT, and time-of-day was found, in any of the groups (*p* > 0.30).

## Discussion

The aim of this study was to investigate the effects of two different cTBS protocols on the SoA in healthy subjects, and to define which protocol induces stronger behavioral changes.

Our results showed that the SAS decreased after cTBS over the rPPC, compared to Vertex stimulation, eliminating potential confounds due to unspecific effects of stimulation at whole-brain level. This effect was visible only in the 30-Hz protocol with an effect size which can be considered large (Cohen, 1988b), whereas 50-Hz cTBS over the rPPC did not show significant changes in the SAS compared to the control region. Based on the power analysis, which showed that 15 subjects are already sufficient to elicit behavioral effects with our study design, and on the CIs of the effects, the lack of significance in the 50-Hz protocol is unlikely driven by a small sample size or type II error.

### The 50-Hz and the 30-Hz Protocols

Two main characteristics distinguish the two protocols: the stimulation parameters, at 50 Hz with stimulation pattern at 5 Hz, instead of 30 Hz with stimulation pattern at 6 Hz, and the number of pulses, 600 in one single application instead of 1620 over two 15 min apart applications.

The difference in the stimulation pattern has been studied by recording MEP (Goldsworthy et al., 2012a). At neurophysiological level, small variations in the stimulation parameters may affect the effectiveness of the protocol. For instance, the introduction of short 8-s breaks after 2 s of stimulation within the same session, such as in iTBS, induces excitatory effects, in contrast to the inhibitory ones of cTBS (Huang et al., 2005). Our results are in line with previous research, showing that cTBS at 30 Hz induced stronger effects, compared to cTBS at 50 Hz (Goldsworthy et al., 2012a).

The second difference between the protocols was the overall number of pulses and sessions. The literature on this topic has not established an optimal protocol yet. One study showed that several sessions of cTBS at 30 Hz, at 15-min interval from each other, induced stronger inhibition at behavioral level than one single application (Nyffeler et al., 2006b). Others have reported that single 50-Hz cTBS applications did not elicit neurophysiological changes, whereas paired cTBS ones at 10-min intervals did (Goldsworthy et al., 2012b). Our results support these findings, and their interpretation may come from a consolidation mechanism of synaptic plasticity. Animal research has shown that spontaneous neuronal activity, as a result of random sensory inputs due to the ambient or inputs from other brain regions, can eliminate the effects of cTBS-induced synaptic modifications (Zhou et al., 2003), which could be the reason why one single application of cTBS did not elicit significant changes. Synaptic modifications can be stabilized by a second cTBS application appropriately spaced (Abraham, 2003; Nyffeler et al., 2006a). Moreover, the duration of the breaks between sessions has been found to play a crucial role in the effectiveness of TBS. If the second session is delivered after shorter breaks, up to 5 min, it may interfere with the building up of this mechanism and reverse the effects, whereas stimulation after longer breaks, over 20 min, may find cortical activity back to baseline levels (Gamboa et al., 2011), and fail to engage synaptic plasticity. Within this framework, our results suggest a 15-min break as a good choice for the duration of the interval.

It was out of the scope of this study to measure the specific contributions of the stimulation parameters, but overall, our results confirm that lowering the stimulus frequency to 30 Hz and shifting the stimulation pattern toward the higher part of the theta range, as well as increasing the number of pulses and introducing a 15-min break between sessions, elicited strong behavioral changes after stimulation of the rPPC. Future neurophysiological studies should assess the contribution of the single stimulation parameters on the overall effectiveness of the TBS protocols.

It is worth noting that the target area may play an important role in the outcome of stimulation. Neurophysiological studies, which rely upon MEP measures, can only be performed on the motor cortex (Jacobs et al., 2014), and this limits the generalizability of their results. Behavioral studies can be performed on different brain areas, but their results depend on higher cognitive functions, which are sensitive to environmental parameters, such as expectations on motor outcome, as in the present study (Edwards et al., 2012). Our results align with previous literature on behavioral effects of NIBS, where cTBS has been used to modulate, for instance, the PPC (Cazzoli et al., 2009), the prefrontal cortex (Ott et al., 2011), Broca’s area (Clerget et al., 2011), and the cerebellum (Picazio et al., 2013). Future research should expand the validity of our findings in cognitive domains other than agency processing, and confirm the efficacy of our cTBS protocol. To this end, other techniques have been proposed to study the effects of TMS on the brain, such as fMRI (Eldaief et al., 2011; Halko et al., 2014) or EEG (Farzan et al., 2016) (for a complete overview, see Pascual-Leone et al., 2011). Their application may be a valuable tool to investigate the susceptibility of neural networks to NIBS.

Regarding safety, none of the subjects reported long-lasting side effects of stimulation. The mild headache reported by some participants after the 30-Hz protocol may be due to the overall length of the experiment, about 20 min longer than the one with 50-Hz cTBS, or to the itching sensation of the pulses, more than double compared to the 50-Hz one, on the skin.

### Modulation of the Sense of Agency and Its Clinical Relevance

The rPPC, in particular the rTPJ, is part of the agency network (Zito et al., 2020b), and its inhibition leads to decreased SoA in healthy subjects (Zito et al., 2020a). Our results showed promising results in stimulating the rTPJ as abnormal SoA has been reported in various neurological conditions including schizophrenia, Gilles de la Tourette syndrome, or functional neurological disorders (FND) (Edwards et al., 2013; Delorme et al., 2016). In addition, an abnormal activity of the rTPJ has been consistently found in FND (Aybek and Vuilleumier, 2016). Restoring normal activity patterns by inhibiting the rTPJ with 30-Hz cTBS has the potential for a treatment option in these disorders. However, the direction of the behavioral effects of stimulation needs further investigation: Our results showed that inhibition of the rPPC decreased the SAS. In line with the concept of “virtual lesion” (Ziemann, 2009), a speculative explanation of this finding is that, when the rPPC is inhibited, its capacity to fairly judge mismatches between intended and executed action is impaired, and this in turn may generate an exaggerated feeling of being out of control, i.e., a lower SAS. Future research should measure the activity of the rPPC before and after cTBS, and correlate the behavioral effects of stimulation with specific patterns of neural activity linked to the SoA.

### Limitations

This study presents some limitations. The first one is the fact that we did not compare the effects of the protocols in the same individuals. However, we showed that the baseline levels of the two groups were not statistically different, and it is then unlikely that our findings were driven by group differences. Similarly, it is unlikely that the stimulation intensities, age, and time-of-day influenced the outcome, as no correlation was found between these variables and the difference post–pre cTBS in the SAS. One potential limitation is the difference in the number of pulses and applications between the protocols, which makes a comparison at neurophysiological level hard. However, our focus was on the behavioral outcome, and our choice was driven by the existing literature, where the 30-Hz protocol has been often applied in consecutive sessions (Fu et al., 2015; Nyffeler et al., 2019; Zito et al., 2020a), in comparison to the 50-Hz one, originally developed for one single application. Another limitation may be the measurement of the rMT through the visible twitch method, which relies upon the experience of the examiner, and we did not measure MEP. However, our results indicated no correlation between the intensity and the effects of stimulation, and the rMT was always identified by the same examiner, which reduced the possibility of additional variability in its calculation. We did not use online neuronavigation of the TMS coil, as our participants had no brain images available at the time of the experiment. However, previous research has shown that our localization method has high spatial accuracy (Braet and Humphreys, 2006; Busan et al., 2012; Zanon et al., 2013), and that a large area of a few square centimeters within the rTPJ is responsible for agency processing (Miele et al., 2011; Zito et al., 2020a). Given the 5 cm^2^ focality of the butterfly coil used in this experiment (Deng et al., 2013), we decided that higher accuracy achievable with neuronavigation was not necessary.

## Conclusion

In this paper, we studied behavioral effects of two cTBS protocols applied over the rPPC in healthy subjects. We showed that two applications of 30-Hz cTBS with 6 Hz stimulation pattern elicited behavioral changes in the SoA, whereas one single application of 50-Hz cTBS protocol did not. Our results may be driven by a consolidation mechanism of synaptic transmission. Our findings bring valuable information, as they show that the application of the 30-Hz protocol over the rPPC is safe and able to elicit strong behavioral effects on the SoA. This may be relevant for future studies aiming at modulating activity of the rPPC in cognitive domains other than agency, and in patients affected by impaired agency, who could benefit from rehabilitation strategies based on NIBS (Ridding and Rothwell, 2007).

## Data Availability Statement

The raw data supporting the conclusions of this article will be made available by the authors, without undue reservation.

## Ethics Statement

The studies involving human participants were reviewed and approved by the Cantonal Ethics Committee of Canton Bern, Switzerland. The patients/participants provided their written informed consent to participate in this study.

## Author Contributions

GZ, YW, J-CL, and RM conceptualized and organized the research project. GZ, JK, JB, and SW executed the research project. GZ and JK contributed to statistical analysis—design and execution and manuscript—writing of the first draft. GZ, YW, J-CL, RM, and SA contributed to statistical analysis—review and critique. GZ, YW, J-CL, JK, JB, SW, RM, and SA contributed to manuscript—review and critique. All authors contributed to the article and approved the submitted version.

## Conflict of Interest

The authors declare that the research was conducted in the absence of any commercial or financial relationships that could be construed as a potential conflict of interest.

## Abbreviations

aMT: active motor threshold
APB: abductor pollicis brevis
CI: confidence interval
cTBS: continuous theta burst stimulation
FND: functional neurological disorders
iTBS: intermittent theta burst stimulation
JoA: judgment of agency
JoP: judgment of performance
MEP: motor evoked potentials
MNI: Montreal Neurological Institute
NIBS: non-invasive brain stimulation
rmANOVA: repeated measures analysis of variance
rMT: resting motor threshold
rPPC: right posterior parietal cortex
rTMS: repetitive transcranial magnetic stimulation
rTPJ: right temporo-parietal junction
SAS: summary agency score
SoA: sense of agency
TBS: theta burst stimulation.
